# Faculty Perspectives on Teaching Death and Dying: Exploring Personal Benefits, Challenges, & Lessons Learned

**DOI:** 10.1093/geroni/igaf122.1418

**Published:** 2025-12-31

**Authors:** Autumn Decker, Raven Weaver, Cory Bolkan

**Affiliations:** Rede Group, Pullman, Washington, United States; Washington State University, Pullman, Washington, United States; Washington State University, Vancouver, Washington, United States

## Abstract

Faculty are expected to carefully navigate sensitive and complex topics related to death and dying, often prioritizing students’ comfort over their own. This creates space for students to reflect on their own beliefs, values, and attitudes toward mortality, fostering resilience and promoting compassionate care in both personal and professional contexts. However, less is known about faculty comfort with the topic. Through content analysis of secondary data collected using a phenomenological research approach, we explored faculty perspectives on teaching death and dying. This paper highlights their personal lessons learned to inform others teaching similar courses in U.S.-based higher education settings. Key informant semi-structured interviews (n = 27) yielded insights into common benefits, challenges, and effective teaching strategies. More specifically, three key themes emerged regarding faculty health and well-being: (1) the importance of engaging in formal therapy and counseling; (2) having a strong support system (e.g., family, friends, colleagues); and (3) investing in meaningful self-care for both them and their students. Participants also stressed that faculty teaching these courses must be deeply invested in ensuring students receive the best and safest experience when engaging with sensitive material. They also emphasized the importance of boundary setting and referring students to school counseling services when needed. Lastly, participants recommended sharing university, local, and national resources, including opportunities for mental health first aid training, with instructors. These lessons learned offer valuable insights into best practices and are crucial for advancing death education on a broader scale.

